# Consensus on Quality Indicators of Postgraduate Medical E-Learning: Delphi Study

**DOI:** 10.2196/mededu.9365

**Published:** 2018-04-26

**Authors:** Robert Adrianus de Leeuw, Kieran Walsh, Michiel Westerman, Fedde Scheele

**Affiliations:** ^1^ Athena Institute for Transdisciplinary Research VU University Amsterdam Amsterdam Netherlands; ^2^ VU University Medical Center Amsterdam Netherlands; ^3^ British Medical Journal Learning British Medical Association London United Kingdom

**Keywords:** postgraduate medical education, continuing medical education, e-learning, distance education, quality tool, quality indicators, education, medical, education, medical, continuing, education, distance

## Abstract

**Background:**

The progressive use of e-learning in postgraduate medical education calls for useful quality indicators. Many evaluation tools exist. However, these are diversely used and their empirical foundation is often lacking.

**Objective:**

We aimed to identify an empirically founded set of quality indicators to set the bar for “good enough” e-learning.

**Methods:**

We performed a Delphi procedure with a group of 13 international education experts and 10 experienced users of e-learning. The questionnaire started with 57 items. These items were the result of a previous literature review and focus group study performed with experts and users. Consensus was met when a rate of agreement of more than two-thirds was achieved.

**Results:**

In the first round, the participants accepted 37 items of the 57 as important, reached no consensus on 20, and added 15 new items. In the second round, we added the comments from the first round to the items on which there was no consensus and added the 15 new items. After this round, a total of 72 items were addressed and, of these, 37 items were accepted and 34 were rejected due to lack of consensus.

**Conclusions:**

This study produced a list of 37 items that can form the basis of an evaluation tool to evaluate postgraduate medical e-learning. This is, to our knowledge, the first time that quality indicators for postgraduate medical e-learning have been defined and validated. The next step is to create and validate an e-learning evaluation tool from these items.

## Introduction

E-learning, which also goes by many other names, is taking up a strong position in medical curricula because of its flexibility, richness, and potential for resource sharing and for high value in light of its cost [1]. E-learning is suggested as an eligible instrument for interprofessional learning [2], and Goh described e-learning not as just hype, but as a core aspect of medical education in the future [3].

However, the debate on what denotes good-quality e-learning is ongoing. More explicitly, the lack of knowledge on what constitutes good-quality e-learning has been identified as one of the main inhibitors of its usefulness [4]. Cook postulated that e-learning is not always cheaper or more efficient than traditional forms of medical education. However, he also stated that e-learning can be a very important innovation when it becomes “low-cost, low-tech, but instructionally sound ‘good enough’ online learning” [5]. The problem is that there is no useful model for “just good enough” postgraduate medical e-learning. The literature shows that there are no specific working models for this target audience [6] and that the models and tools that are used are diverse. We have previously provided a list of quality indicators [6] and tried to find the underlying constructs of which items are important and meet the needs of learners [7]. In this way, we tried to provide the categories necessary to evaluate postgraduate e-learning. Both for educators involved in postgraduate e-learning and for users themselves, it is crucial to know that e-learning is worth their investment in it. Previous research showed that users are less motivated and less eager to undertake an e-learning module when they are in doubt about its quality [7]. Furthermore, experts believe that it is necessary to know what quality features are required and expected of an e-learning course before it is created [7].

In response to this debate on what constitutes good-quality medical e-learning, we set out to provide an empirically based set of quality indicators. Thus, we performed a Delphi procedure to evaluate suggested quality indicators from the literature. To our knowledge, this study is the first international consensus by both educational experts and experienced users on quality indicators in postgraduate medical e-learning.

## Methods

In this study, we performed a Delphi procedure to determine consensus on the possible quality indicators for e-learning in postgraduate medical education.

### Study Design

Escaron et al describe the Delphi method as being well suited to informing health education [8]. It is based on the concept of pooled intelligence and should enhance the individual judgments and capture the collective opinion of experts [9]. We performed the Delphi digitally, facilitated by RAD, because online Delphi studies reduce costs, time, and effort [9] and are not limited by geographical boundaries. The downside is that participants have a consultative role and disagreements are hard to explore. This is even more the case when using a digital medium to communicate. To maximize the effectiveness of the Delphi, we followed the guidelines of de Villiers et al [9]. We first provided a definition of e-learning to the expert panel, then started with a questionnaire of items. After analyzing the results, we removed items without consensus, added comments on the remaining items, and, if applicable, added new items.

### E-Learning Definition

For this Delphi we chose the following, slightly adapted definition from Sangrà et al: “E-learning is an approach to teaching and learning, representing all or part of the educational model applied, that is based on the use of electronic media and devices as tools for improving access to training, communication and interaction and that facilitates the adoption of new ways of understanding and developing learning” [10]. To simplify the discussion, we chose to talk about stand-alone, asynchronous, and distant e-learning (and not learning management systems). We provided all participants with this definition and an explanation in the introduction of the Delphi.

### Expert Panel Selection

For this study, we used 2 expert groups: medical educators and end users. Medical educators are experts in the theory and practice of creating e-learning and end users know what it’s like to use the e-learning in their daily practice. A suitable expert is defined in the literature as someone who possesses the relevant knowledge and experience and whose opinions are respected by fellow workers in their field [9]. For this study, we defined an educational expert as a member of a national medical education platform (usually a university- or government-led foundation aimed at improving and validating medical education) or someone who has been published in peer-reviewed international journals on the subject of medical e-learning, and who has had at least 3 years’ experience with medical education and e-learning development. We defined experienced postgraduate users as postgraduate residents who graduated at least 2 years ago and who have had exposure to e-learning throughout their postgraduate training.

We selected experts by means of an inquiry to the National Education Board in the Netherlands and from author contacts. We invited experienced users in the Netherlands and Great Britain because we had local contacts there. An expert panel usually consists of 15 to 30 participants, with 5 to 10 participants per category [9]. Our aim was to have 10 experts and 10 experienced users but, as we believe that educational experts have a better background in the theoretical grounding of education, we preferred to have a few more educational experts on the panel. We thus aimed for 13 experts and 10 users [9].

### Questionnaire Development

**Figure 1:**
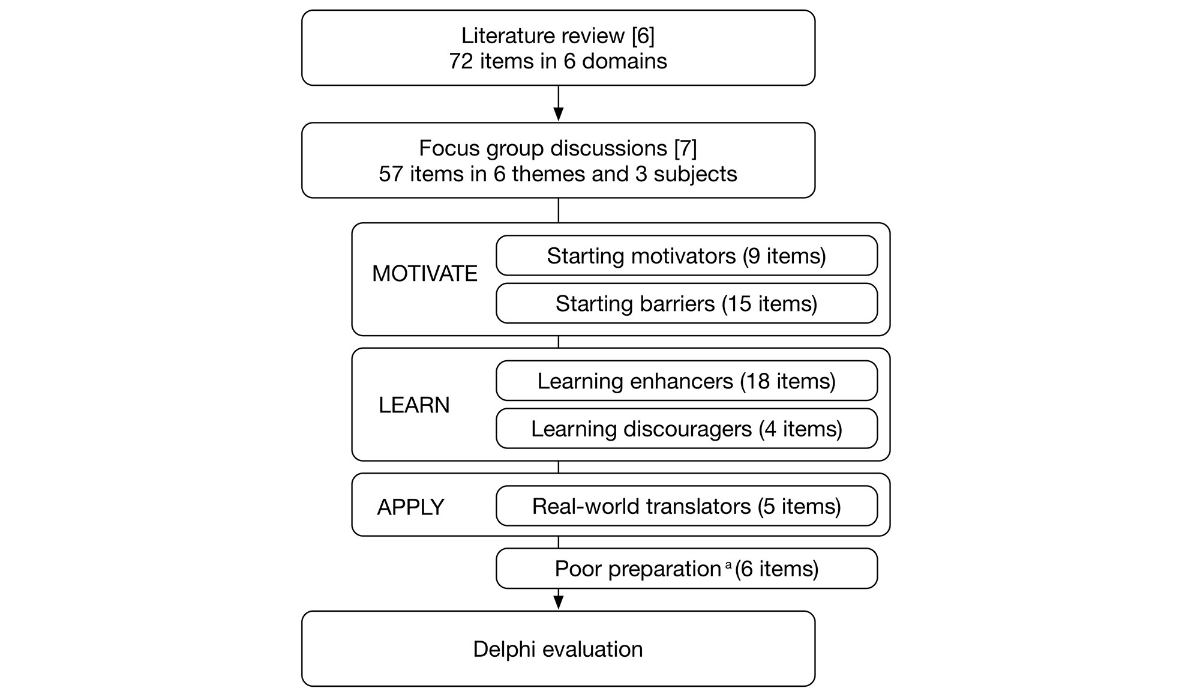
Subjects and themes of postgraduate medical e-learning quality indicators.^a^The preparation theme is aimed at e-learning authors only.

The initial set of indicators was based on 2 previous studies and contained quality characteristics from the literature [6] (72 items) and from focus group discussions (resulting in 57 items) with both experts and end users [7] (see Figure 1). These previous studies gave a total of 57 items in 6 themes on 3 subjects: motivate, learn, and apply. The subject *motivate* consisted of indicators that increase the learner’s level of motivation in the theme, called starting motivators, and indicators that form a barrier to starting or finishing the e-learning, called starting barriers. The next step was the subject *learn*, which consisted of all pedagogical indicators that either facilitate (learning enhancers) or limit (learning discouragers) the learning experience. The subject *apply* was made up of indicators that help the learner to translate and apply the e-learning into their daily practice (real world translators). Finally, the theme poor preparation (6 items) consisted of indicators that help an author prepare for the creation of an e-learning resource. Items such as “Plan a feasible budget to prevent incompletion of the e-learning due to lack of funds” were not originally aimed at the end user and therefore evaluated only by the experts.

The questionnaire started with introductory text explaining the subjects, providing a definition of e-learning, and asking the experts and users to imagine e-learning that was “just good enough” and targeted at medical postgraduates. After that, the experts and end users evaluated the individual items on a 5-point Likert scale and were able to add comments [9].

After we agreed on the content of the questionnaire, we performed a pilot round with 5 participants (2 educators and 3 end users). After incorporating their feedback on the items, we invited the experts to fill out the questionnaire digitally. We started the first round with 57 items.

### Statistical Analysis

After each round, we worked out consensus by calculating the rate of agreement: (agreement – disagreement/agreement + disagreement + indifferent) × 100%. We used a rate of agreement of two-thirds to accept an item. An item was rejected when there was no consensus after 2 rounds, or when an item was rejected by a rate of agreement lower than –66% in the first round (the rate of agreement scale ranges from –100 to 100). There is no consensus in the literature regarding the best rate of agreement to be used; the range used has been between 51% and 80% [11]. We chose to use two-thirds as proposed by de Villiers et al [9].

The Ethical Review Board of the Association for Medical Education gave ethical consent (file number 475), after which all participants gave their written informed consent.

## Results

We sent the first invitation emails out on March 19, 2017, and received the final response on July 20, 2017. We invited 23 experts, of whom 13 replied and participated, 9 did not reply to the invitation, and 1 did not consider himself an expert on postgraduate medical e-learning. We invited 17 experienced users, of whom 5 did not reply, 2 could not participate due to other obligations, and 10 were able to participate. In total, we had 23 participants, of whom 23 responded in both rounds. Of the participants, 13 (57%) were male. The average age of the experts was 49 years and that of the users was 31 years. The experts came from the Netherlands (n=7), Great Britain (n=3), Canada (n=2), and South Africa (n=1). They had an average of at least 3 years’ experience creating or evaluating medical e-learning and together had published 29 articles. A total of 4 were members of the Dutch Association for Medical Education expert group on e-learning. The users were Dutch (n=7) and British (n=3), and had more than 3 years’ experience as residents, and had attended on average more than 2 e-learnings during their residency.

**Figure 2:**
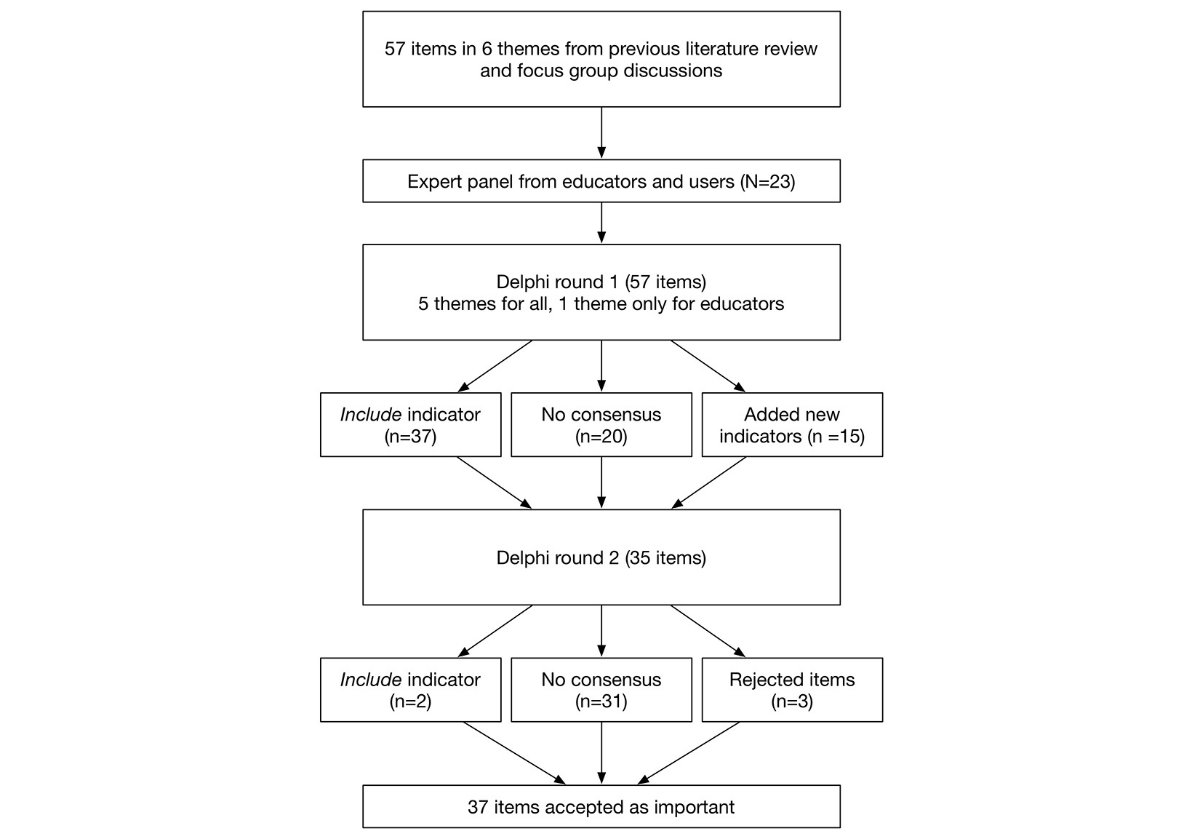
Flowchart of the Delphi results.

In the first round, 37 items were accepted as important, with a rate of agreement of above two-thirds. No items were rejected, there was no consensus on 20 items, and 15 new items were added by the participants (Figure 2). In the second round, we added the comments from the first round on the items without consensus and added the 15 new items (35 items in total). We also added 3 explorative questions based on comments from the first round, exploring the usefulness of a list of indicators. Multimedia Appendix 1 shows all items, rate of agreement, and consensus.

The first explorative question was “Do you think it is possible to define a minimum and general set of criteria that can be generalized for all types of medical e-learning?” A total of 17 participants thought this was possible, 5 were not sure, and 1 thought it was too complicated. Worries about such a list of indicators included the following:

But I would be concerned that to be applicable for all types of medical e-learning it might be too general and therefore not practically useful

Medical educator 1

Yes, but it’s like evidence-based medicine: you must be able to deviate with motivation.

Medical educator 4

The experts also raise the concern of a fast-changing definition of e-learning:

the term e-learning is in a fast-changing technological world with different needs and skills for makers (and for users) and is difficult to define—without maker- or user-focused definition and context

Medical educator 1

e-learning doesn’t mean anything in particular, tech can be used in every aspect of med-ed, and lots of different tech can be used for different purposes….

Medical educator 5

Participants mentioned that which form of e-learning these indicators are about is very important to explain.

The second explorative question was “Do you think a 10-question survey, like the one mentioned in the introduction, would be of added value to the current evaluation tools?” It was thought by 14 (64%) to be of added value, 7 (32%) were not sure, and 1 (4%) thought it was not of added value. Arguments were

...it would help setting priorities

Medical educator 8

...general design principles probably will apply to e-learning as well. So why the need of a specific tool? I think there may be added value in evaluating the specific additive value of technology. But I am not sure. That’s why I am participating in this Delphi.

Medical educator 3

The third explorative question was to explore how many items participants considered to be workable. The general opinion was “the less the better, but as much as needed”. When asked for a number, participants responded with a range of numbers from 10 to 20.

We then evaluated the remaining 35 items (see Multimedia Appendix 1). There was consensus that just 2 items should be included, 3 were rejected, and there was no consensus on the rest. After this round, a total of 72 items were addressed and, of these, 37 were accepted and 34 rejected (see Table 1).

**Table 1:** The final quality indicators. Items 32-37 are expert theme preparation items.

Subject and item	
**Motivate**	
	1. Create a feeling of importance within the learner
	2. Create a feeling of responsibility within the learner
	3. Provide enough time to complete the e-learning
	4. Define the purpose of the e-learning (knowledge, skills, and behavior or attitude)
	5. Formulate the learning objectives and preferably visualize them
	6. Provide an overview of all content
	7. Prevent concerns about the quality of the content
	8. Do not force, although obligation might be possible
	9. Create the feeling that the learner is being taken seriously
	10. Use a flexible platform, so that the content can be modified by the educator
	11. Provide easy accessibility from all locations and devices
	12. Use easy and clear navigation
	13. Use a simple layout with a sitemap
	14. Software should be safe and secure
	15. Access should be fast
	16. Make clear which device is needed and advise the learner about the skills needed
**Learn**	
	17. Enable the learner to personalize the module
	18. Allow nonlinear learning
	19. Show what has already been achieved and what has not yet been done (progress bar)
	20. Provide technical support
	21. Add summaries
	22. Give feedback
	23. Add exercises and assignments
	24. Create interaction with the content
	25. Do not stress or frustrate the learner
	26. Avoid nonadaptive content
	27. Do not create too distractive a design or learning activities
**Apply**	
	28. Make the content translatable to the real world
	29. Update and maintain the e-learning
	30. Provide sources of information and keep access available after the course is finished
	31. Evaluate the e-learning after the course and collect feedback
	32. Know your target audience and adapt learning objectives accordingly
	33. Identify the authors at the beginning of the e-learning
	34. Create a timeline with objectives and expectations of the production stage
	35. Form a development team with at least 1 content expert, 1 educational expert, and 1 information technology expert, and let them all commit a certain amount of time before starting the development
	36. Plan a feasible budget to prevent incompletion of the e-learning due to lack of funds
	37. Consider an appropriate learning environment and learning management system

## Discussion

### Principal Findings

We performed an international Delphi study with educational experts and experienced users that led to 37 quality indicators for postgraduate medical education. To our knowledge, this is the first list of quality indicators for postgraduate medical e-learning with an evidence-based foundation: first selecting all the indicators mentioned in the literature, then adding to this list by focus group discussions, and finally selecting the items using a Delphi.

Cook et al wrote in 2009 that internet-based learning is associated with a positive effect, but that future research should directly compare different internet-based interventions [12]. Developing peer-reviewed training and guidelines for e-learning should also be the foundation of academic e-learning [13]. However, to compare e-learning or e-education methods and to guide authors, we need to provide them with a tool. These indicators should form the basis for such an e-learning evaluation tool that can help to compare different types of education with e-learning. To evaluate the effect of e-learning in postgraduate medical education, we need a list of indicators. We believe that these indicators should be supported by experts in the field and the final end users of the e-learning resources. This study produced such a list.

After the first round of the Delphi, the experts expressed the challenges of an evaluation of this type. The term *e-learning* can be confusing, the *added value* to a landscape of many other evaluation tools might be limited, and the indicators may be *too general*. The term e-learning, as discussed in the introduction, is broad. However, when it is well defined, we believe it can still be a workable term. There are many quality models in the literature [14], and e-learning has been evaluated many times [15]. But these models are aimed at different target audiences, the origin of the indicators is ill defined, and the validation is limited, when present at all. The final indicators from our study are quite generic and are difficult to translate back to postgraduate learning. It could very well be that the items identified in this study are applicable to graduates or other groups of learners.

### Limitations and Strengths

Potential pitfalls in Delphi studies are the imposition of preconceptions on respondents and poor techniques for summarizing and presenting the group response. We tried to limit these pitfalls by producing a simple and straightforward questionnaire. Participant selection was limited to those who responded and, by choice, from the countries of the authors’ residence. Therefore, our study lacked a certain cultural diversity, making the results possibly less generalizable.

The strength of the final indicators lies in the balance of general aspects of evaluation and the specifics added when needed. We believe that the 6 themes (motivation, barriers, learning enhancers, learning discouragers, real-life translation, and poor preparation) are general enough to be applied to all kinds of e-learning.

### Conclusion

Creating e-learning for postgraduates is not enough; evaluation and improvement should not be additional but mandatory to ensure maximum effect. E-learning quality indicators can be sorted into 3 groups (motivate, learn, and apply) with 5 general themes (motivators, barriers, learning enhancers, learning discouragers, and real-life translators) and a list of items that can be used in preparing e-learning resources.

This study provided a list of quality indicators for postgraduate medical e-learning. This list is unique in its evidence-based foundation and in the way that it applies broad themes with specific indicators. The most logical next step is to create and validate an evaluation tool based on these indicators.

## Appendix

Multimedia Appendix 1 Delphi results per question and per round.
