# Supplementary material for: Consensus on Quality Indicators of Postgraduate Medical E-Learning: Delphi Study
Source: JMIR Med Educ. 2018 Apr 26;4(1):e13. doi: 10.2196/mededu.9365 (PMC5945990; doi:10.2196/mededu.9365)
Supplement: Multimedia Appendix 1 [file mededu_v4i1e13_app1.pdf]

Appendix A. Delphi results per question and per round

	Round 1 RoA[a]				Round 2			
**Question**	**Sum**	**EDUC**	**USERS**	**Result**	**Sum**	**EDUC**	**USERS**	**Result**
**Theme motivators and barriers**								
Q1a. The e-learning should create a feeling of importance within the learner	90	85	100	Accepted				
Q1b. The e-learning should create a feeling of importance within the learner by encouraging them to complete it by a realistic deadline, after which the e-learning modules should no longer be accessible	19	-7	71	Undecided	-5	40	-56	Undecided
Q2. The e-learning should create a feeling of responsibility within the learner	67	57	86	Accepted				
Q3. The authors' authority and expertise should be identified	43	50	29	Undecided	-18	-15	-22	Undecided
Q4. The e-learning should provide a reward when finished	52	50	57	Undecided	-36	-31	-44	Undecided
Q5. The learner should be provided with enough time to complete the e-learning	90	93	86	Accepted				
Q6. The educator should give a definition of the purpose of the e-learning (knowledge, skills and/or behaviour/attitude, defining the e-learning objectives for the next section	90	86	100	Accepted				
Q7. The educator should formulate the learning objectives and preferably visualise them	95	92	100	Accepted				
Q8. There should be different levels of learning within the e-learning (basic, novice and advanced)	19	29	0	Undecided	-9	8	-33	Undecided
Q9. The e-learning should provide an overview of all content (with details such as what you can learn, where to learn it, etc.)	81	79	86	Accepted				
Q10. Users of the e-learning (learners) should not have concerns about the quality of the	95	100	86	Accepted				

e-learning content								
Q11. The learners should not be forced to undertake the e-learning, although compulsory e-learning might be possible	67	73	57	Accepted				
Q12. The learner should have the feeling that they are being taken seriously (e.g. you should involve stakeholders, aim at the expected level of experience, not make the visuals too childish, etc.)	86	93	71	Accepted				
13.1 The availability of offline e-learning should be ensured	33	14	71	Undecided	18	0	44	Undecided
13.2 E-learning should be flexible, so that the content can be modified by the educator	76	71	86	Accepted				
13.3 There should be a search function	38	43	29	Undecided	18	8	33	Undecided
13.4 There should be easy accessibility from all locations and devices	62	43	100	Undecided	77	69	89	Accepted
13.5 Multi-tasking with other platforms, e.g. to play music, should be allowed	24	0	71	Undecided	14	0	33	Undecided
13.6 There should be easy and clear navigation	100	100	100	Accepted				
13.7 There should be a simple layout with a sitemap	86	79	100	Accepted				
13.8 Software should be safe and secure, e.g. by installing an SSL certificate (for secure communication over the internet)	67	57	86	Accepted				
13.9 Access should be fast; therefore, content should be aptly sized and formatted (smallest possible image size, etc.)	76	64	100	Accepted				
Q14. The educator should make clear what device is needed for the e-learning and the educator should advise the learner about the skills needed	85	92	71	Accepted				

Q15. The e-learning should have added value over the available learning content (offer new material or be presented in a different way)	60	46	86	Undecided	-18	-38	33	Undecided
added new items								
Q242. You should add the previous users' ratings of the module				Added	30	50	0	Undecided
Q243. You should try to have the e-learning referred to as an important source of information by, e.g., tutors, educators, etc.				Added	-42	-27	-63	Undecided
Q244. Materials should include a date to indicate relevance				Added	-33	-25	-44	Undecided
Q245. You should formulate and communicate an “expiry date” for the e-learning, after which it should be re-evaluated and, if needed, updated				Added	-86	-83	-89	Rejected
Q246. A digital medium could in itself already be of added value over a book, so the content itself does not have to be				Added	-50	-30	-83	Rejected
**Theme learning enhancer**								
Q17a. The e-learning should enable the learner to personalise the module (save and continue, pre-testing, create ownership)	95	100	86	Accepted				
Q17b. The e-learning should enable the learner to personalise the module by allowing a-linear learning (linear is fixed from A to Z, while a-linear allows users to skip parts, return to them and create their own learning route)	71	64	86	Accepted				
Q17c. The e-learning should enable the learner to personalise the module by showing what has already been achieved and what has not yet been done (progress bar)	100	100	100	Accepted				
Q18. The e-learning management system should provide technical support	67	57	86	Accepted				

Q19. The e-learning should provide horizontal communication (communication between learners) as well as being an essential part of the learning objective	-15	-15	-14	Undecided	50	54	44	Undecided
Q20. The e-learning should provide vertical communication (communication between learner and educator) as well as being an essential part of the learning objective.	0	-7	14	Undecided	55	54	56	Undecided
Q21. The e-learning should be based on the concepts of problem-based learning as much as possible	45	15	100	Undecided	5	15	-11	Undecided
Q22. The e-learning should provide a minimum set of contents needed to achieve the learning objectives	62	64	57	Undecided	-20	9	-56	Undecided
23.1 Summaries	71	64	86	Accepted				
23.2 Repetition of content in different media/formats	5	21	-29	Undecided	-5	-8	0	Undecided
23.3 Feedback	95	93	100	Accepted				
23.4 Exercises and assignments	100	100	100	Accepted				
23.5 Interaction with the content	81	93	57	Accepted				
23.6 Multimedia use per Mayers Cognitive Load principles of Multimedia	43	50	29	Undecided	9	38	-33	Undecided
23.7 Self-assessment	52	50	7	Undecided	45	46	44	Undecided
23.8 Reminders by e.g. email for e.g. an assignment or unfinished module	29	0	86	Undecided	27	15	44	Undecided
23.9 Activate/stimulate the learner by using fun elements, challenges or serious gaming	48	43	57	Undecided	32	31	33	Undecided
23.10 Do you have any comments or ideas concerning a learning activity?		-7	-43	Undecided	9	0	22	Undecided
24. The e-learning should not be perceived by the learner as too long; in other words, the e-learning should be as short as possible and not make in-depth information mandatory	62	57	71	Undecided	-19	8	56	Undecided

Q25. The e-learning should not stress or frustrate the learner (no negative feedback from a supervisor or punishment for failure, a generous time window for access)	68	58	86	Accepted				
Q26. Non-adaptive content should be avoided	95	92	100	Accepted				
Q27. The e-learning should not be too distractive in its design or learning activities (prevent distractions like irrelevant images or moving elements)	75	62	100	Accepted				
added new items								
Q247. All presenters should be native speakers in the language, or have as neutral accents as is possible. Unlike in face-2-face, misunderstandings in video presentation cannot easily be corrected. If it is not possible to find a native speaker, the video should be subtitled and proofed by native speakers				Added	-57	-42	-78	Rejected
Q248. If possible, create a an evidence-based layout (e.g. font style), based on a guideline provided by a checklist like this one				Added	-6	0	-16	Undecided
Q249. The e-learning should be easy for students with a disability (dyslexia)				Added	-52	-42	-67	Undecided
Q250. Materials should record user actions for analysis				Added	-35	-42	-25	Undecided
Q251. Other learners' performance should be recorded and shown to new users to motivate them				Added	46	69	11	Undecided
Q252. Fun elements can create a childish feeling, which should be avoided.				Added	14	15	11	Undecided
**Theme apply**								
Q29. Make the content translatable to the real world by using examples from the daily	100	100	100	Accepted				

practice of the learner and recognisable subjects, adding relevant items like time management only when applicable.								
Q30. Update and maintain the e-learning	100	100	100	Accepted				
Q31. Provide sources of information outside the e-learning and keep access available after the course is finished	76	79	71	Accepted				
Q32. Evaluate the e-learning after the course and collect feedback for the following topics: user experience, effectiveness, usability and costs.	67	79	43	Accepted				
**Expert theme preparation**								
Q33. Know your target audience and adapt learning objectives accordingly	100			Accepted				
Q34. Identify the authors at the beginning of the e-learning. The authors should (personally) be known to the educators, and information from an unknown source should not be used	67			Accepted				
Q35. Create a timeline with objectives and expectations of the production stage	70			Accepted				
Q36. Form a development team with at least 1 content expert, 1 educational expert and 1 IT expert, and let them all commit a certain amount of time before starting the development	80			Accepted				
Q37. Plan a feasible budget to prevent incompletion of the e-learning due to lack of funds	100			Accepted				
Q38. Consider an appropriate learning environment/learning management system	100			Accepted				
Added new items								
Q253. Each module should contain story telling/a narrative; it does not have to be "cases" or				Added	-24	-15	-37	Undecided

"PBL" - all sorts of stories can be engaging, e.g. from different points of view (patient, doctor, relative), historical stories, stories of scientific discovery relevant to the learning								
Q254. Use the TPACK framework (http://www.tpack.org/). It is possible that. e.g., one person is both educational expert and IT expert				Added	7 (8**)	25	-16	Undecided
Q255. Create a team of creators, who are also the users of the e-learning. In doing so, you will keep contact with your audience and can spread the message much more easily				Added	-43	-38	-50	Undecided
Q256. Change the theme name "preparation" to "production process of e-learning"				Added	22	29	14	Undecided

[a] RoA = Rate of Agreement
